# A Fluorescence-Based Wireless Capsule Endoscopy System for Detecting Colorectal Cancer

**DOI:** 10.3390/cancers12040890

**Published:** 2020-04-06

**Authors:** Mohammad Wajih Alam, Seyed Shahim Vedaei, Khan A. Wahid

**Affiliations:** Departement of Electrical and Computer Engineering, University of Saskatchewan, Saskatoon, SK S7N 5A9, Canada; shahim.vedaei@usask.ca (S.S.V.); khan.wahid@usask.ca (K.A.W.)

**Keywords:** wireless capsule endoscopy, fluorescence, colorectal cancer, non-invasive

## Abstract

Wireless capsule endoscopy (WCE) has been widely used in gastrointestinal (GI) diagnosis that allows the physicians to examine the interior wall of the human GI tract through a pain-free procedure. However, there are still several limitations of the technology, which limits its functionality, ultimately limiting its wide acceptance. Its counterpart, the wired endoscopic system is a painful procedure that demotivates patients from going through the procedure, and adversely affects early diagnosis. Furthermore, the current generation of capsules is unable to automate the detection of abnormality. As a result, physicians are required to spend longer hours to examine each image from the endoscopic capsule for abnormalities, which makes this technology tiresome and error-prone. Early detection of cancer is important to improve the survival rate in patients with colorectal cancer. Hence, a fluorescence-imaging-based endoscopic capsule that automates the detection process of colorectal cancer was designed and developed in our lab. The proof of concept of this endoscopic capsule was tested on porcine intestine and liquid phantom. The proposed WCE system offers great possibilities for future applicability in selective and specific detection of other fluorescently labelled cancers.

## 1. Introduction

The wireless capsule endoscopy (WCE) system is becoming increasingly popular for examining the entire human gastrointestinal (GI) tract, as this method eases patients’ discomfort and pain compared to other endoscopy methods [1]. However, there are still many challenges (such as image quality, frame rate, battery life, automatic detection of abnormalities, localization, etc.) associated with this promising technology, and these limit its use in modern day healthcare system [2,3]. These tiny swallowable capsules are designed to reach the areas where access is limited without performing surgery. However, the frame rate and image quality of the currently available WCE devices are insufficient for optimal screening [4]. 

**Figure 1:**
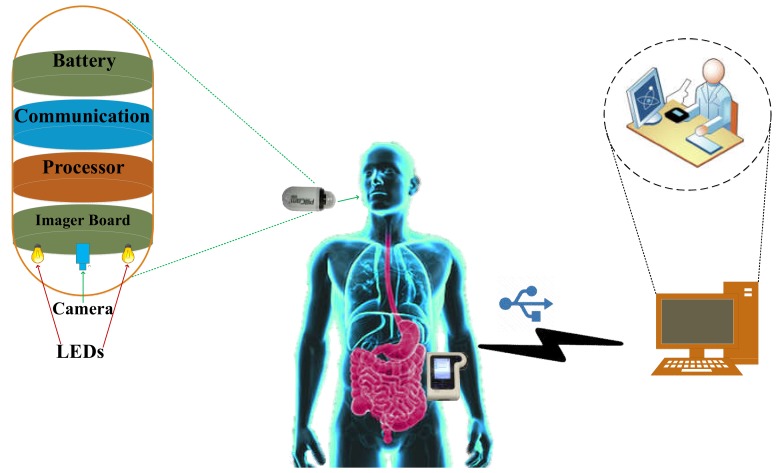
Illustration of a typical wireless capsule endoscopy (WCE) system.

A typical WCE system consists of a pill-shaped electronic capsule, a data recorder with radio frequency (RF) antenna, and a workstation computer with software, as shown in Figure 1. The capsule is integrated with an image sensor, illumination source, batteries, communication module, and other components [5,6]. Once the capsule is active and swallowed by the patient, it starts capturing images and sending the captured images to the data recorder via the RF transmitter. The data recorder (which is usually attached to the patient’s waist using a strap) receives and saves the data. After 24 h, the patient returns the data logger to the physician who then downloads the images from the data logger to a computer, where the images are examined using the vendor’s software [7]. The software labels the frames suspected to include abnormalities, which would then be examined by the physician [7]. This entire process is labor-intensive and time-consuming [8]. The accuracy of the detection software and human error make this technology prone to diagnostic errors. Such errors can also be a result of inexperience, lack of training and subjective interpretation of the data [9], hampering the wide acceptance of this technology. Although the conventional WCE system, which uses white light imaging, can now provide high-resolution images, proper diagnosis depends on the characteristics captured by the capsule, which varies due to morphological changes of the GI wall [10]. Such morphological changes often appear very late, thus hampering the overall diagnostic as well as prognosis.

Colorectal cancer is the third commonest type of cancer diagnosed in both men and women. It is also the third commonest cause of cancer-related deaths in the United States [11]. About 1 million new cases of colorectal cancer are diagnosed each year, among which about 72% of cases arise in the colon, and the remaining 28% in the rectum, which collectively make it the leading cause of cancer-related deaths worldwide. The stage at which the cancer is first diagnosed is an important factor in determining the prognosis and survival of patient [12]. Early detection of cancer is essential for improving the survival rate of cancer patients [13]. There are different state-of-art methods that are currently used in the healthcare system for diagnosing colorectal cancer: computed tomography colonography [14,15], flexible sigmoidoscopy [16,17], double contrast barium enema [18], stool DNA test [19], fecal occult blood testing [20], and the colonoscopy (the gold standard) [21]. Although these state-of-art methods are popular among physicians, they are not very comfortable for the patients. Moreover, the patients are afraid of invasiveness, pain and embarrassment, which makes them not want to go through the conventional procedure unless it is entirely necessary. This effects early detection adversely. Despite a tremendous advancement in therapeutic and diagnostic possibilities, colorectal cancer in patients are often detected in the advanced stage, and the survival rate for such patients is only 14%. However, if the same cancer was diagnosed at an early stage, the chances of survival can increase up to 90% [22]. 

Fluorescence imaging is a technique which is widely used in medicine, biology, and biochemistry. It is the process which visualizes fluorescent dyes or proteins as labels to study the biological molecules and phenomena [23]. The visualization of these tissues are usually achieved by labelling the proteins via a nanobody, antibody or bio-specific ligand [24]. Fluorescent labelling [25] binds fluorescent dyes covalently to biomolecules, such as proteins, so that they can be differentiated from non-fluorescent (non-bound) biomolecules. Fluorescence can be detected by using autofluorescence or specific labelling of proteins. It has been used to detect various physiological abnormalities for many decades as a non-invasive procedure. For instance, fluorescein has been used to detect ophthalmic pathologies since 1960. This method has also proven to be useful in successful detection of cancer. Attaching the fluorophore to a targeting agent enables the functionality of fluorescence imaging in the clinical environment, thereby increasing the specificity and sensitivity of detection. The combination of fluorophores with targetable cancer biomarkers have led to increased interest of both researchers and the medical industry in the area of fluorescence-guided surgery. Clinical trials were first introduced in early 2010. They use various fluorophores along with a range of targeting strategies (such as, antibodies, molecules, peptides, activatable fluorophores and multimodal fluorophores). 

Autofluorescence is the process of detecting fluorescence emissions arising from endogenous fluorophores. Various tumor types are detectable both by autofluorescence techniques as well as by the use of exogenous fluorescent markers [26]. Tumor-specific fluorescent imaging agents [27] help surgeons with real-time intraoperative feedback on the location of tumors. The accurate detection or demarcation between tumor, inflammation and normal tissue is often difficult due to the lack of visual distinction among them. Targeted fluorescence imaging is therefore helpful in this regard, and can help solve this problem by providing real-time tumor detection or visualization [28]. Thus, conjugating fluorescent dye to specific tumor-recognizing ligands (such as peptides or antibodies) enhances the specificity of tumor detection considerably. Various tumor-specific agents have already shown feasibility in early phase clinical trials. It is important to choose biomarkers which express strong fluorescence several fold higher than the surrounding normal tissues in order to provide better selectivity and sensitivity. Previously, we have proposed a low-cost fluorometer which worked on the principle of fluorescence to detect breast cancer cells [29] and colorectal cancer cells [30]. 

In this work, a complete WCE system is designed based on the principle of fluorescence to detect colorectal cancer. This system has potential to automate the detection process and improve the overall efficiency and accuracy of diagnosis. 

## 2. Materials and Methods 

**Figure 2:**
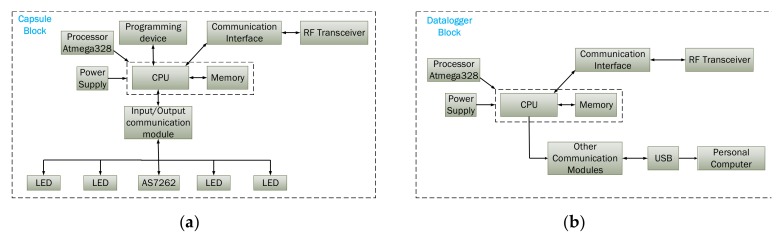
Hardware blocks of the proposed capsule prototype: (**a**) capsule block (**b**) data logger block.

**Figure 3:**
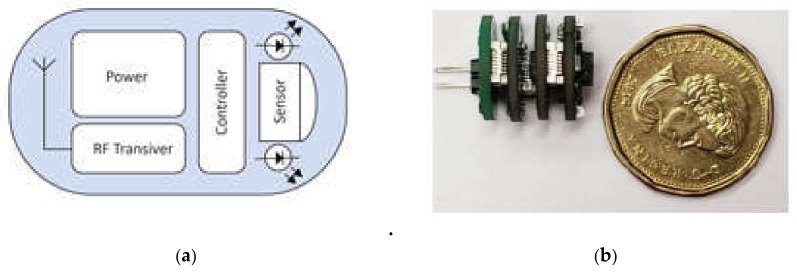
(**a**) Internal components; (**b**) capsule prototype with 4 printed circuit board (PCB) layers (compared with a CAD $1 coin).

The developed capsule prototype consists of the following blocks: an optical block, a microcontroller, telemetry, and a power module. The hardware diagram of the proposed system is shown in Figure 2. Figure 3a shows the internal components of the capsule. Figure 3b shows the 4-layered printed circuit board (PCB) design of the prototype. The components were chosen so that the capsule size remains small while optimizing the power consumption, performance, and sensitivity. 

**Figure 4:**
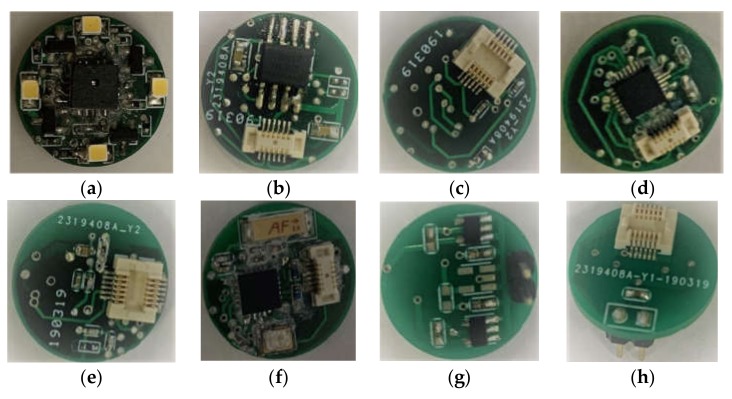
Illumination with spectral sensor: (**a**) top view; (**b**) bottom view. (ii) Control/processing unit: (**c**) top view; (**d**) bottom view. (iii) Telemetry unit: (**e**) top view; (**f**) bottom view. (iv) Power unit: (**g**) top view; (**h**) bottom view.

**Figure 5:**
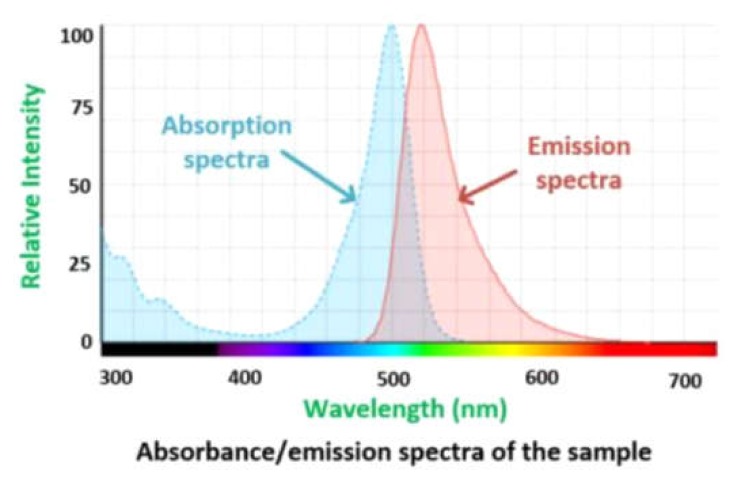
Absorption/emission spectra of the sample adapted from [31].

The proposed device consists of four main electronic printed circuit boards (PCBs): a sensor board with illumination components, a control/processor board, a telemetry board and the power module as shown in Figure 4. The placement of each electronic component and its associated connections was crucial in this miniaturized design. The four PCBs are connected via headers, and the design was selected as the best solution for the compact, efficient, and reliable design. Figure 4a shows the top layer which consists of four light emitting diodes (LEDs) arranged around the spectral sensor. These LEDs works in the ultraviolet (UV) range having a peak emission wavelength of 395 nm and excitation wavelengths ranging between 380 nm and 410 nm (Kingbright, ATS2012UV395). The sensor used for sensing the fluorescent light is an AS7262 spectral sensor manufactured by ams AG. It has 6-channel multispectral sensing capabilities, and works in the visible range between 430 nm and 670 nm with a full-width half max (FWHM) of 40 nm; i.e. 450 nm, 500 nm, 550 nm, 570 nm, 600 nm and 650 nm, each with a 40 nm FWHM. Moreover, each wavelength channel has a Gaussian filter characteristic. The spectral sensor is connected with the microcontroller via the I2C protocol. When the microcontroller is initialized at startup, the sensor is also ready to capture the spectrum. At each iteration, the microcontroller selects the wavelength channel in operation, and captures the data from the sensor accordingly. In our case, the peak excitation and emission wavelength of fluorescein is 494 nm and 521 nm respectively as shown in Figure 5. As such, the green channel is selected for the detection. 

A comprehensive analysis was performed before selecting the complete illumination system. The choice of these components depends upon several factors, but trade-offs were considered between power consumption, miniature size, and the light intensity that would be required for the capsule to perform efficiently. The chosen LED is 2 mm × 1.25 mm in size. Furthermore, the absorption/emission spectra of the target fluorophore were considered while selecting these components. The spectral sensor is situated in the middle of the LEDs, equidistant from all LEDs. The electrical connections with the second layer (microcontroller) were achieved through pins 23 and 24 via a 11 × 2 header that helps the board to correctly align with the optical sensor. Each LED is driven via digital pulse width modulation (PWM) pins of the microcontroller, which will help to control the brightness, if needed. Figure 4a,b show the sensor/illumination unit of the capsule. The control board is the heart of the capsule as it controls the processes and connects all other PCB layer. The microcontroller used in this device is an ATmega328P. This microcontroller can operate at between 1.8 and 5.5 volts. The chip has 8 analog input pins and 14 digital I/O pins, which makes interfacing with other components easy. This microcontroller receives the data from the spectral sensor and sends it to the RF transceiver.

The capsule prototype uses an nRF24L01 + radio transceiver for wireless communication. This transceiver has a good range and can communicate for a considerable distance through walls with negligible data loss. The nRF24L01 + module operates at 2.4 GHz. At this working frequency, the wireless transmission module shows the characteristics of having long transmission range and strong penetrability, which is helpful for WCE application. This module is an important bridge for wireless communication (both transmission and reception) with the external data logger. The power board contains miniature low dropout regulators (LDO), each having a rated voltage of 3.3 V and current of 150 mA. Any kind of silver oxide or Li-ion battery with a voltage range over 3.7 V could be used to power up the capsule prototype. 

**Figure 6:**
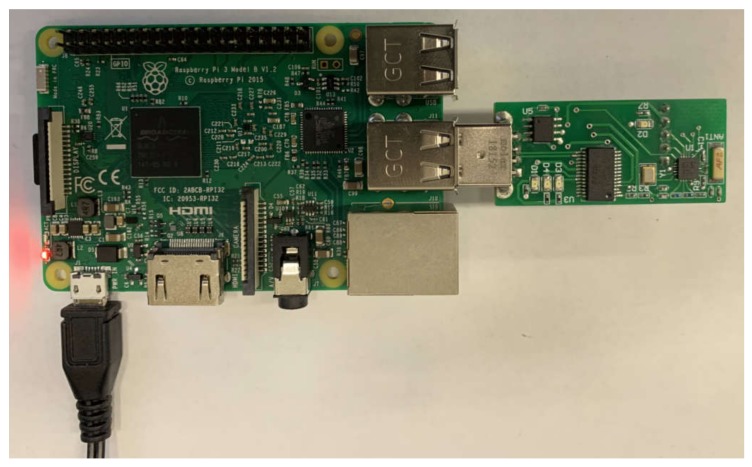
Data logger connected to the Raspberry Pi system.

The data logger is based on an Atmel microcontroller, which is capable of receiving the capsule’s signal and transmitting the received data to a computer. It has two modes of operation. First, when the data logger is plugged into a computer via a USB port, it detects the “software mode” operation, which means that the data logger can receive signals from the capsule and transmit them to the computer in real-time. On the other hand, when the data logger is powered up by an external power source without having connection to the computer, the device enters a “stand-alone mode”. In this mode, the data logger will still receive data from the capsule, but stores the received data in the internal memory and transmits them to the Raspberry Pi board for further processing. By connecting the data logger to the computer, our software can read the data from the memory. Figure 6 shows the data logger which is connected to the Raspberry Pi system. 

## 3. Results and Discussion

### 3.1. Screening Intestine

**Figure 7:**
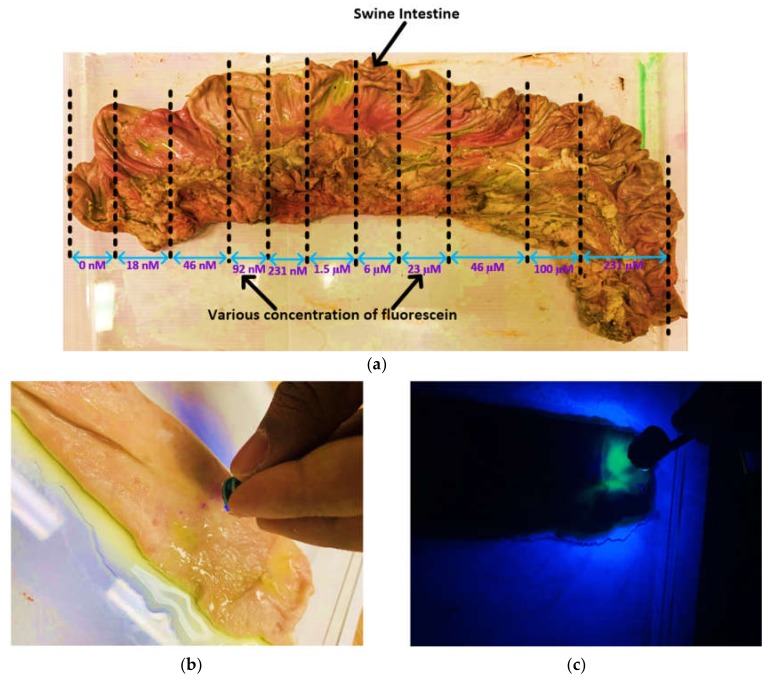
Screening of a porcine intestine by the capsule prototype: (**a**) various concentrations of fluorescein solution sprayed over the intestine; (**b**) demonstration of the capsule in working conditions; (**c**) demonstration of the capsule in working conditions when no external light is present.

One of the major objectives of this work is to demonstrate the ability to detect the increasing levels of fluorescence emitted by the cancer cells compared with normal cells. Fluorescein is a safe-dye and commonly used in vivo. It has found increased use in many applications, such as: ophthalmology and neurosurgery [32,33]. Fluorescein has also been used to differentiate between cancer cells and normal cells during intraoperative surgery for different types of cancer [27,34,35]. The fluorophore can be administered into body via different pathways: intravenously [34,36], intradermally [35], or by spraying [37]. The fluorescence intensity may vary depending on the region where the cancer is located, the time since it was injected, and on the fluorophore concentration. A fluorescein concentration of 5 mg/kg (~1.5 µM) to 20 mg/kg (~6 µM) is enough for detectable fluorescence emitting from the cancer cells when injected intravenously [38,39]. In this work, different concentrations of fluorescein were prepared (231 µM to 18 nM) in order to test whether the proposed capsule can detect different levels of fluorescence. Moreover, the functionality was also tested on a porcine intestine (shown in Figure 7a). Figure 7b and c show the experimental setup when the external light is on and off, respectively. As can be seen in Figure 7c, only the area with fluorescein glows when exposed to ultraviolet (UV) light from the developed prototype. 

**Figure 8:**
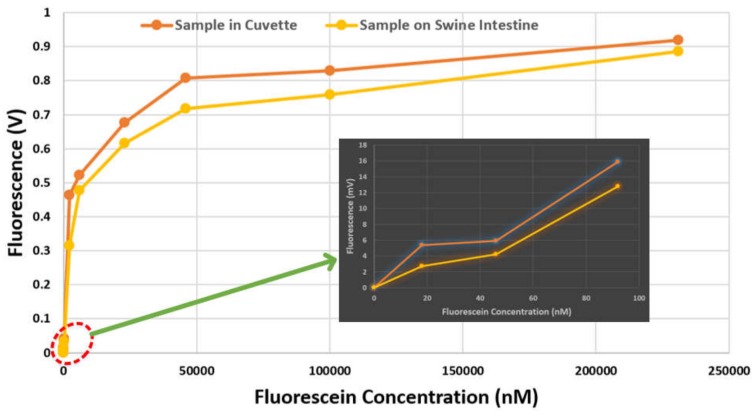
Change of fluorescence intensity with varying fluorescein concentrations. Inset: zoomed plot when the concentration is low.

Figure 8 shows the Fluorescein intensity captured by the capsule prototype for varying concentrations. The fluorescence intensity increases with increasing concentration and the device is also able to detect the concentration that emits a low level of fluorescence. It can be seen that the response from the tissue with no fluorescein and the area with fluorescein were clearly distinguished even at a low concentration (see inset of Figure 8). Hence, in an area of intestine with a high concentration of the biomarker (i.e., resembling cancer), a good response or signal will be found, whereas in an area without the biomarker, no fluorescence signal will be detected. Moreover, if the fluorescein is conjugated with an antibody that targets a specific antigen found in colorectal cancer cells [40], targeted fluorescence-based WCE is possible. We have presented work based on this principle in previous studies [29,30,41].

**Figure 9:**
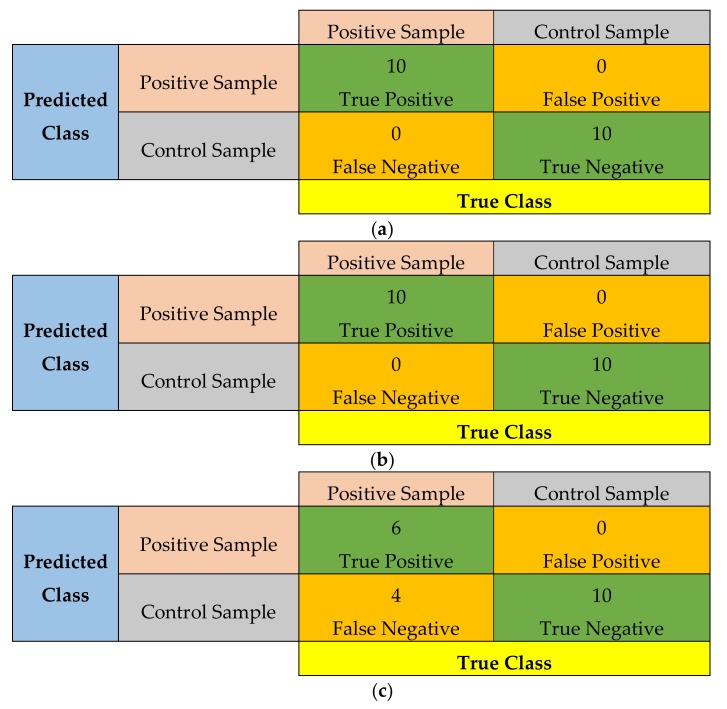
Confusion matrix from samples of: (**a**) high concentration, 231 µM; (**b**) medium concentration, 1.5 µM, and; (**c**) low concentration, 231 nM.

**Figure 10:**
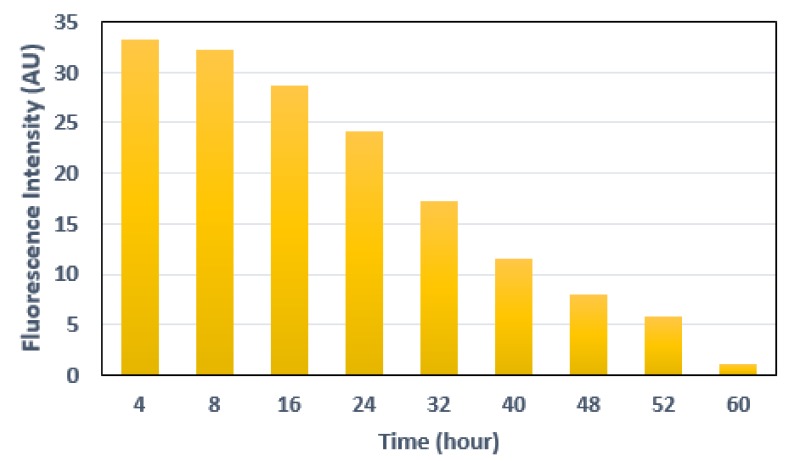
Fluorescence intensity measured at different time-points from the initial application of fluorescein.

In order to further assess the performance of the proposed WCE device, we used the following four indices: sensitivity, specificity, accuracy, and precision, as expressed below:

(1) $$Sensitivity=\frac{TP}{TP+FN}$$

(2) $$Specificity=\frac{TN}{TN+FP}$$

(3) $$Accuracy=\frac{TP+TN}{TP+TN+FP+FN\;}$$

(4) $$Precision=\frac{TP}{TP+FP}$$

where, TP is the number of true positives, TN is the number of true negatives, FP is the number of false positives, and FN is the number of false negatives. The samples were divided into three categories: 231 µM (high concentration), 1.5 µM (medium concentration) and 231 nM (low concentration). Each category contained 10 positive samples (i.e., area sprayed with fluorescein) and 10 control samples (i.e., area without fluorescein). Figure 9 shows the confusion matrix using the measurements. It can be seen that the prototype is able to detect the samples accurately when the fluorescein concentration varies from high to medium level. However, when the concentration was low, the results are not conclusive. The sensitivity, specificity, accuracy and precision indices for 231 µM and 1.5 µM were 1, 1, 1 and 1 respectively, and for 231 nM were 0.6, 1, 0.8 and 1 respectively. To further assess the potential use of fluorescein for the detection of tumors, the fluorescein was sprayed over the porcine intestine and the signal was measured at various time-points with 1.5 µM solution. The fluorescence intensity at different time-points is shown in Figure 10. For WCE applications, the fluorescence signal should be present for about 8 h.

### 3.2. Data Logger

When the capsule is turned on, it starts to sense the signal through the sensor module (AS7262), and begins to capture the data and send to the data logger via RF transceiver. The data logger receives and stores the data. The software on the data logger is a Python script which communicates through the serial port. Operational parameters, such as spectrum capture interval and capsule illumination mode can be controlled via the software. Table 1 shows the breakdown of the material costs of our system. The miscellaneous cost in Table 1 includes packaging, 3D-printing and fabrication costs. Note that labor and engineering costs are not included in the table. 

### 3.3. Power Consumption

Table 2 shows the current consumption of the individual components in both active and inactive modes. For an average of 14.85 mA current consumption, the proposed device can run for 12 h with batteries of 178.2 mAh capacity [42]. The runtime can be extended by connecting two or three batteries in series. Therefore, the lifetime is enough to cover most patient’s GI transit time [43,44]. However, an increased battery life may be useful for patients who need a longer GI transit time (capsule retention or other chronic disease) [45,46]. 

### 3.4. Wireless Communication

There are several wireless transceivers available, each having their pros and cons depending on the application. A Nordic transceiver is used in the proposed system. One of the major challenges in wireless communication is the possibility of data corruption during transmission and reception. The Nordic transceiver has a built-in capability for cyclic redundancy check (CRC)-based error detection and retry with acknowledgement (i.e., it resends the data packet until it is successfully received), which makes it a reliable choice. However, several retries may hamper the data transmission rate in a noisy environment [47]. 

**Figure 11:**
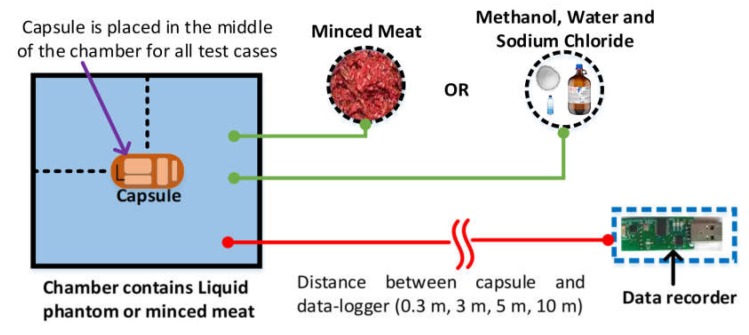
Experimental setup for testing transmission rate of the proposed prototype. A plastic storage box is used as a test chamber.

**Figure 12:**
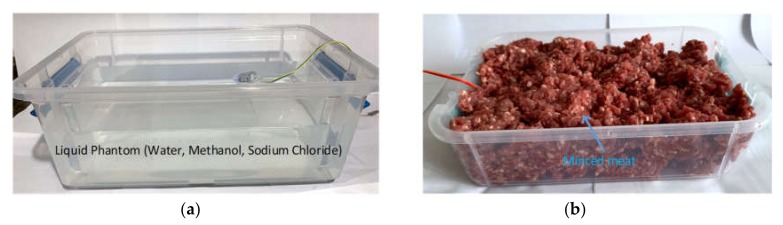
Measurement setup to test the transmission rate of the proposed capsule in: (**a**) liquid phantom; (**b**) minced meat.

In order to verify the performance of our prototype further at 2.4 GHz [48,49], we performed two additional experiments using an equivalent liquid phantom and minced meat as shown in Figure 11. Since the nRF24L01 module has a CRC functionality (i.e., detects error bits automatically), it is not possible to measure the exact BER (bit error rate, the ratio of the number of bits received in error to the total number of bits received) in this experiment. However, considering the loss of the entire packet, we have implemented test cases to measure the number of bytes lost per test case. The results are shown in Table 3. The prototype was placed in a liquid phantom first, and then in 1.8 kilogram of minced meat (as shown in Figure 12). The liquid phantom was made from pure water, methanol and sodium chloride to mimic human GI fluid [50,51]. The chamber size of liquid phantom and minced meat were: 40 cm × 30 cm × 16.5 cm (length × width × height) and 26 cm × 17 cm × 7 cm (length × width × height) respectively. The capsule was placed in the middle of the chamber in all test cases. The distance between the capsule and the data logger was varied gradually from 0.3 to 10 meters, and data from the capsule were sent to the data logger. All data bits were transmitted to the data logger with no loss when the distance between the data logger and the capsule in liquid phantom was up to 5 meter. When the distance increased to 10 meters, we started to notice loss of data packets and the transmission rate was found to be 90%. In another test case, the capsule was placed in minced meat. As before, when the distance between the capsule and the data logger was varied up to 5 meters, no loss of data packets was observed. However, when the distance increased to 10 meters, we noticed that transmission rate was affected, with 80% of data successfully transmitted. Since, the data logger is wearable and generally worn around the waist in practice, the distance between a swallowed capsule and the data logger is generally about 0.3 meter [52]. Therefore, we expect our prototype to work at 2.4 GHz spectrum with no loss of data during communication.

### 3.5. Comparison with Other WCE Systems

Table 4 shows a comparison with different WCE systems which are available both commercially or are at the research stage. This table is divided into two categories: fluorescence-based capsules and non-fluorescence-based capsules. As can be seen from the table, the commercial WCE systems are non-targeted endoscopy. Most of them are camera-based, and target the small bowel region, and lack functionality for automatic detection of abnormalities. As a result, once the data is unloaded with the help of vendor’s software on a computer, post-processing is required to find the frames of interest. This requires significant amount of time and labor. Furthermore, the use of camera makes the capsule expensive both cost-wise (>$500) and power-wise, ultimately affecting the battery life and its affordability. 

Moreover, the frame rate and resolution of the GI image in the existing system is not optimal for proper diagnosis of GI abnormalities. There are few non-camera based WCE systems which can measure different physiological parameters, such as pH, temperature and pressure. Removing the cameras will significantly increase the battery life of the capsule, and reduce the cost. In addition, there are other capsules at the research stage which aim to detect bleeding and automate the detection of a cancer. Some of them utilize the property of autofluorescence to automate the detection process. However, autofluorescence comes with reduced specificity and sensitivity in automating cancer detection. As a result, this technique does not provide enough contrast when compared to normal and abnormal cells, because the signal from the cancerous cells are often buried under the autofluorescence of healthy cells. In addition, the intensity of autofluorescence is dependent on diet [72]. To the best of our knowledge, there is no work which proposes fluorescence-based targeted WCE system for detecting colorectal cancer. 

## 4. Summary and Outlook

Targeted fluorescence-based WCE systems have the advantage of improving specificity and sensitivity over the current generation of conventional WCE systems. They improve the diagnostic as well as screening procedures of the entire GI tract. In addition to being non-invasive, these capsules present us with an opportunity to enable automatic detection of abnormalities. One of the main challenges in designing such a system is to make these devices small enough that a patient can swallow it. The components used to build the proposed prototype are available off-the-shelf. The developed capsule size is 16 mm × 34 mm. Further miniaturization can be done by using custom-designed components. Moreover, this device also removes the need for bulky components (such as optical filters and a mounting holder) which reduces the cost of the device.

Most colon cancers begin as pre-cancerous polyps. Therefore, screening for cancer in a timely manner will allow physicians to find and treat different types of cancer at an early stage before they cause symptoms. This will lower the burden on both the patient and the healthcare system. After the screening procedure is completed and a positive sign of cancer is suspected, other confirmatory tests (such as CT, PET-CT, MRI, or ultrasound scans) can be performed to confirm the findings and locate the cancerous cell [73]. The follow-up treatment usually depends upon location, stage and type of the cancer. Moreover, researchers have been working on monoclonal antibodies that can recognize and attach to specific proteins which are produced by cells. Each monoclonal antibody can target only a specific protein. Different antibodies differ in functionality and are selected based on the type of protein that they are targeting. Some of these antibodies block the protein that aids in the growth of the cancer, while others detach cancer cells from the blood supply, prohibiting growth [74]. The main treatments for early-detected cancers are surgery and chemotherapy. Other treatments include radiotherapy and targeted cancer drugs [75]. These treatment strategies are based on the stage at which the cancer is detected. 

Due to the invasive characteristics of the traditional endoscopic system, most patients are reluctant to go through the procedure, adversely affecting the early-detection process and the possibility of efficient treatment. Therefore, the non-invasive WCE technology can help with early detection. The proposed device can detect multiple fluorescence signals from multiple fluorophores; therefore, it may be possible to detect other types of cancers by using different fluorophores that would bind to the specific antigen of a specific cancer cell. The development of a single WCE device for detecting multiple cancers may be possible in the future. 

In the future, a camera can also be placed on the other end of the WCE device which will only turn on to capture the GI images when an increased level of fluorescence is detected. This would help the physicians examine the abnormality visually, thereby increasing the efficiency, as well as the reliability of the system. The battery life of the proposed capsule can be improved by using the adaptive illumination technique [76]. Moreover, we have previously tested the PillCam SB3 capsule on horses [77]. It would be interesting to see the application of this technology in veterinary medicine.

In order to provide the best and most effective healthcare, the Internet of Things (IoT) offers new ways of improving the system by presenting novel facilities and enhancing the functionalities of the existing system. It is estimated that the hospital-centered healthcare system will be transformed to a home-centered system by 2030 [78]. Thus, it is imperative that the IoT is introduced into the WCE system as well. The existing WCE systems cannot yet offer real-time detection as the diagnosis is mainly done offline [6]. In addition, a self-data analyzing intelligent system could be helpful, which can select useful data for transmission and save transmission cost, bandwidth, and energy. To develop an IoT-based system, features like intelligence, heterogeneous network connectivity, real-time sensing, and security may be incorporated into the system.

## 5. Conclusions

WCE has enabled a pathway towards pain-free diagnosis and screening of the entire GI tract. This system has encouraged patients to go through the examinations, ultimately paving the way for mass screening and enabling early diagnosis of GI abnormalities. The current generation of WCE systems has several limitations which restrict its wide application. Hence, in this paper, we have presented the design and development of a complete WCE system, which utilized the principle of fluorescence imaging, capable of performing targeted endoscopy. The developed capsule consists of four major units: illumination, processor, transceiver and power. Each of these units was separately tested before integrating into a complete system. Initial experiments with various solutions and phantoms have demonstrated that the proposed device is capable of selective and specific detection of abnormal colorectal cancerous cells. 

## Figures and Tables

**Table 1:** Cost breakdown of the proposed capsule prototype.

Component	Model/Specification	Cost ($USD)
Spectrum sensor IC	ams AS7262-BLGT	3.12
EEPROM IC	AT25SF041-SSHD-T	0.22
LED	ATS2012UV395	1.82
Microcontroller	ATMEGA328P-MMHR	1.8
Transceiver	NRF24L01	2.45
Antenna	ANT-2.45-CHPCT-ND	1.71
PCB		10
Miscellaneous costs		50
	**Total Cost**	**71.12**

**Table 2:** Power consumption of the capsule prototype.

Component	Active Mode	Inactive Mode	Total Current Consumption	Total Power Consumption
Regulators	0.08 mA	0.02 mA	0.08 mA	0.27 mW
Microcontroller	2.53 mA	0.30 mA	2.53 mA	8.35 mW
nRF24L01+	12 mA	0.41 mA	3.13 mA	10.33 mW
LEDs	5.38 mA	0 mA	2.69 mA	8.88 mW
AS7262	6.71 mA	6.32 mA	6.42 mA	21.19 mW
		Total	14.85 mA	49.02 mW

**Table 3:** Transmission performance of the capsule.

Experiment	Distance between Capsule and Data Logger (m)	Transmitted Bytes	Received Bytes	Percentage of Transmitted Data (%)
Liquid phantom	0.3	20	20	100
	3	20	20	100
	5	20	20	100
	10	20	18	90
Minced meat	0.3	20	20	100
	3	20	20	100
	5	20	20	100
	10	20	16	80

**Table 4:** Comparison with other devices: both commercial and research prototypes.

Type	Work	TE	Region of Interest	Resolution	Camera/ Sensor	I/S	TM	RT	Cost of Capsule Only (USD)	DL
**Non-fluorescence based**	PillCam SB [53]	No	Small bowel	256 × 256	1 CMOS	6 LED	RF	Yes	N.G.	Yes
	PillCam SB2 [54]	No	Small bowel	256 × 256	1 CMOS	4 LED	RF	Yes	~530	Yes
	PillCam SB3 [55]	No	Small bowel	256 × 256	1 CMOS	4 LED	RF	Yes	~500	Yes
	PillCam ESO [56]	No	Esophagus	256 × 256	2 CMOS	6 LED	RF	Yes	N.G.	Yes
	PillCam ESO2	No	Esophagus	256 × 256	2 CMOS	2 × 4 LED	RF	Yes	~500	Yes
	PillCam ESO3	No	Esophagus	256 × 256	2 CMOS	2 × 6	RF	Yes	~570	Yes
	PillCam Colon	No	Colon	256 × 256	2 CMOS	2 × 6 LED	RF	Yes	N.G.	Yes
	PillCam Colon2 [57]	No	Colon	256 × 256	2 CMOS	2 × 4 LED	RF	Yes	~550	Yes
	MiroCam [58]	No	Small bowel	320 × 320	1 CMOS	6	HBC	Yes	~380	Yes
	EndoCapsule [59]	No	Small bowel	1920 × 1080	1 CCD	6	RF	Yes	~570	Yes
	OMOM [60]	No	Small bowel	640 × 480	1 CMOS	4	RF	Yes	~380	Yes
	CapsoCam [61]	No	Small bowel	1920 × 1080	4 CMOS	16	USB	No	~380	No
	SmartPill [62]	No	GI tract	N.A.	Pressure, pH & Temperature	None	RF	Yes	~530	Yes
	CorTemp [63]	No	GI tract	N.A.	Temperature	None	RF	Yes	~40	Yes
	VitalSense [64]	No	GI tract	N.A.	Jonah core temperature	None	RF	Yes	~68	Yes
	e-Celsius [65]	No	GI tract	N.A.	temperature	None	RF	Yes	~65	Yes
**Fluorescence based**	Al-Rawhani et al. [66]	No	Small bowel	N.A.	SPAD, ASIC chip	1 LED	RF	Yes	N.G.	Yes
	Demosthenous et al. [67,68]	Yes	Small bowel	N.A.	6 photodiodes	6 laser diodes	SPI	No	~500	No
	Kfouri et al. [69]	No	GI tract	640 × 480	1CCD	8 LED	RF	Yes	N.G.	No
	Nemiroski et al. [70]	No	GI bleeding	N.A.	1 photodiode	1 LED	Zigbee	Yes	~110	No
	Ryou et al. [71]	No	GI bleeding	N.A.	N.G.	N.G.	RF	Yes	N.G.	No
	Proposed device	Yes	Colorectal cancer	N.A.	Spectral sensor	4 LED	RF	Yes	71.12 *	Yes

TE = targeted endoscopy; I/S = illumination source; TM = transmission mode; RT = realtime; ”-” = not mentioned; DL = data logger; N.A. = Not applicable; N.G. = Not Given. * This is the material cost of the lab prototype as shown in Table 1.
